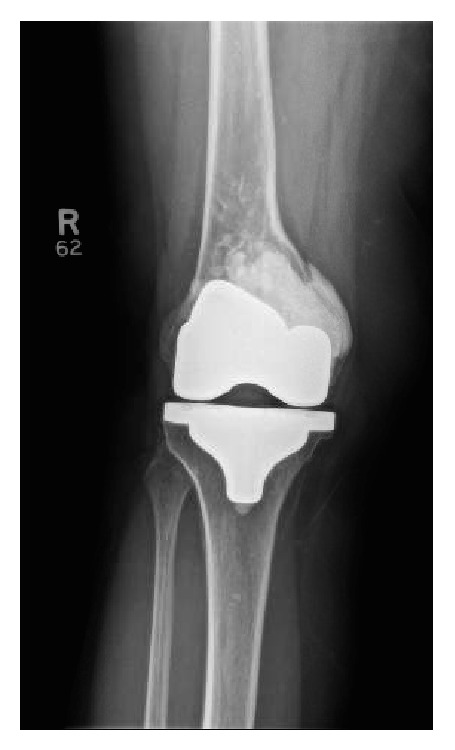# Primary Total Knee Arthroplasty Twenty Years after Distal Femoral Cement Augmentation of a Giant Cell Tumor

**DOI:** 10.1155/2015/283294

**Published:** 2015-04-07

**Authors:** Alejandro Zylberberg, Gillian Bayley, Luca Gala, Paul R. Kim

**Affiliations:** ^1^Division of Orthopedics, Hospital del Trabajador, Ramón Carnicer 185, Providencia Parada de MetroParque Bustamante, Santiago 7501239, Chile; ^2^Division of Orthopedics, The Ottawa Hospital, General Campus, 501 Smyth Road, Ottawa, ON, Canada K1H 8L6

## Abstract

We present a case of knee reconstruction 20 years after treatment of a giant cell tumor (GCT) with curettage and cementation. There is currently an ongoing debate whether cement or allograft bone is the preferred material for filling the void after GCT curettage. In this case we were able to readily implant a primary total knee replacement without disturbing the existing well-interdigitated large cement bolus and did not require any stems or augments for the reconstruction. Given the ease of TKR implantation in this patient, we feel that the use of cement following curettage of a GCT lesion is a better choice than allograft bone which may not provide enough structural support for the knee reconstruction and lead to a much more extensive procedure.

## 1. Introduction

Giant cell tumors (GCT) are locally aggressive benign tumors that typically affect the metaphysis of long bones. They were initially described by Jaffe et al. and represent approximately 15% of benign bone tumors [1]. Treatment options include intralesional curettage with or without chemical adjuvants and cavity filling with either allograft bone or cement. Some patients may develop degenerative changes of the adjacent joint surface following lesion treatment and will eventually require a later reconstructive procedure. We present a case of joint reconstruction performed 20 years after treatment of a GCT with curettage and cementation of the lesion. We were able to utilize a standard primary total knee replacement (TKR) implant without the use of stems or augments. The patient consented to publication of the case.

## 2. Case Report

A 62-year-old female patient was treated in 1991 for a giant cell tumor of the right distal medial femoral condyle with curettage and allograft bone grafting. In the same year, she presented with a local recurrence and had a repeat curettage and cementation of the lesion with polymethylmethacrylate. The patient remained symptom-free for 20 years until she presented to our clinic with increasing knee pain and functional impairment secondary to degenerative change within the knee joint.

**Figure 1:**
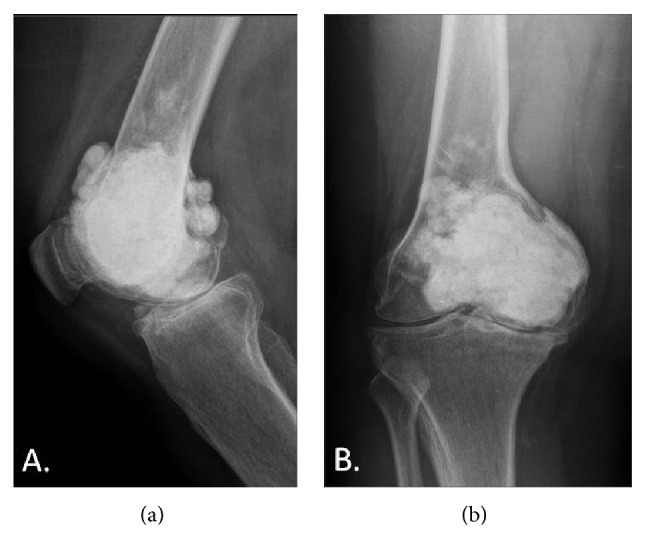


On physical examination, she had a longitudinal anteromedial knee scar with a 10-degree varus deformity. Range of motion was from 15° to 90° of flexion. Plain knee radiographs showed a large bolus of cement occupying the medial femoral condyle, extending superiorly into the metadiaphyseal junction. The joint space showed significant narrowing. There was no evidence of loosening of the cement or tumor recurrence (Figure 1).

The patient was offered a total knee replacement. Different reconstruction options were considered and included a modular oncologic prosthesis and allograft prosthetic composite. However, taking into consideration the good quality of the cement bed, a standard total knee replacement was planned. We had a modular tumor prosthesis available as a backup option.

**Figure 2:**
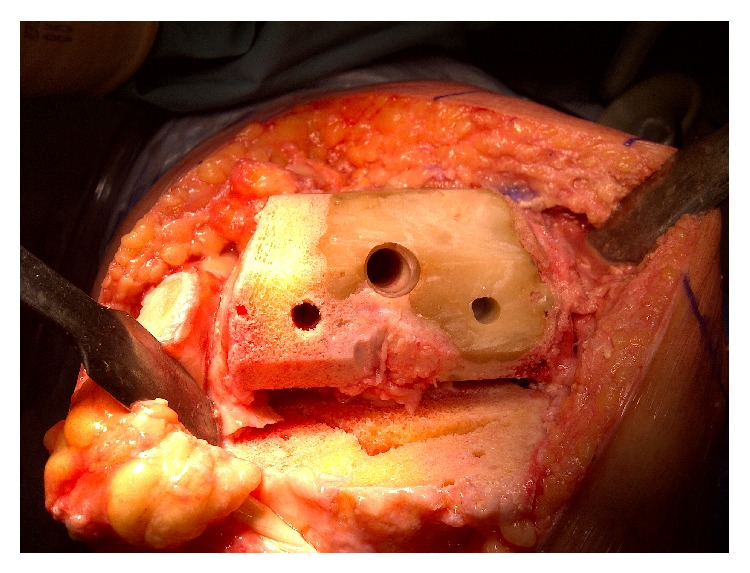


**Figure 3:**
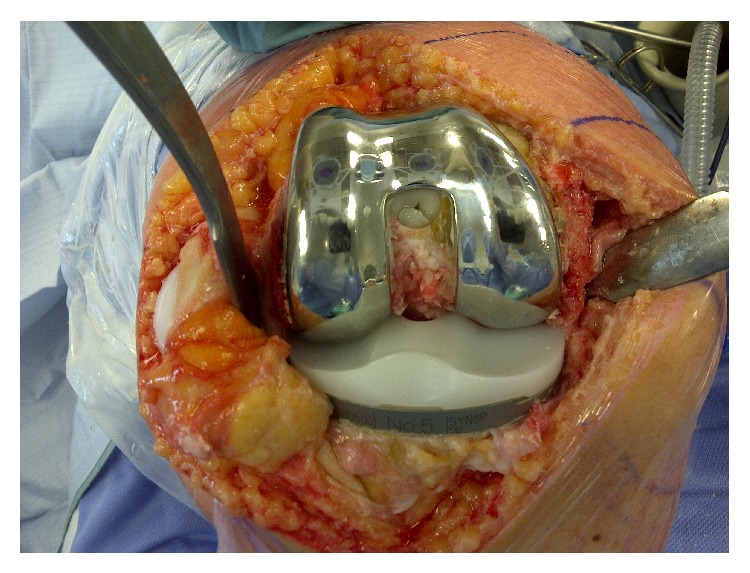


**Figure 4:**
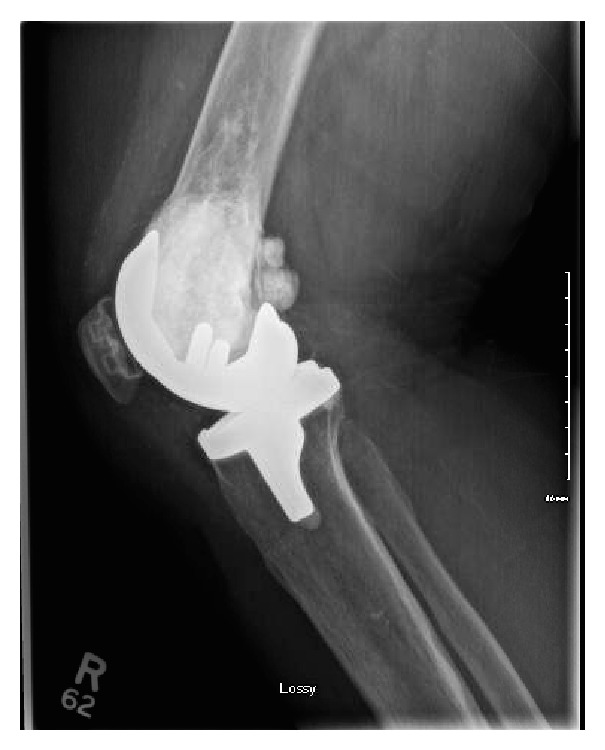


At surgery, severe articular cartilage wear was noted especially in the medial compartment; however, the cement had not yet worn completely through into the joint. The femoral medullary canal was initially opened using a combination of a drill and burr to accommodate a standard intramedullary guide rod. The distal femoral cut was done using a standard saw cutting through the cement without difficulty. The remainder of the femoral cuts were completed after appropriate sizing of the distal femur. The cement remained well interdigitated to the host bone throughout the femoral preparation with no evidence of loosening or fragmentation (Figure 2). Due to the presence of the cement, a cruciate retaining femoral component was selected in order to avoid further cutting into the cement for the box of a posterior stabilized femoral component (Figure 3). The proximal tibia was prepared using standard techniques. The patella was resurfaced. After trialing, final cementation of the definitive components consisting of a Stryker Triathlon size 4 CR femoral component, size 5 tibial baseplate, 9 mm CR insert, and 27 mm patella was completed (all components manufactured by Stryker Orthopaedics, Mahwah, NJ). Postoperative radiographs at a 3-year follow-up showed good component fixation and alignment (Figures 4 and 5). The knee was stable and had a range of motion of 0–100 degrees of flexion.

## 3. Discussion

Giant cell tumors (GCT) are benign, locally aggressive tumors, typically affecting young patients. They commonly present with pain and 10–15% have an associated pathological fracture [2, 3]. The most common presentation site is the distal femur, followed by the proximal fibula. Lesions predominantly occur in the metaphyseal/epiphyseal region but will rarely extend into the joint or capsule [4]. For most cases, curettage with joint preservation is the preferred treatment method [4]. For more advanced disease, block resection and TKR reconstruction have been reported as a good option in primary treatment for limb salvage [5, 6]. With regard to those treated with curettage, aggressive resection of the tumor is important for prevention of recurrence. Trieb et al. [7] suggested that aggressive resection is more important than adjuvant therapy. Other groups have also reported adjuvant therapy to be ineffective; however, the use of adjuvant therapy continues to remain controversial [2, 8–10].

Once the tumor has been adequately resected, structural support of the remaining bone is often necessary [4]. There is ongoing debate whether cement or bone graft is the optimal defect filler. Some studies report good results with bone graft [11, 12], although other groups support the use of cement [13–15] or both graft and cement [16]. Wada et al. [17] reported cement use to be safe for structural support with a low risk of osteoarthritis. Remedios et al. [18] studied 13 patients and reported lower recurrence rates with the use of cement compared to bone grafting. This finding was supported by Kivioja et al. [19], who followed 294 patients over 5 years and also reported lower recurrence rates with the use of cement. van der Heijden et al. published the results of 53 patients treated with curettage and cement analyzing the risk of arthritis development. They concluded that cementation is safe but there was a slightly higher risk of developing osteoarthritis if there was extensive subchondral bone involvement near the articular surface [15]. In a study by Fraquet et al. [20], thirty patients were followed over 6 years. They felt that not only was recurrence diminished, but also a recurrence could be more easily detected if cement was used instead of bone graft. In their review of 38 patients, Kafchitsas et al. [13] also reported a low rate of recurrence and easier recurrence detection with cementation of the lesion.

If an extensive curettage into subchondral bone is required, this could potentially cause destruction of the underlying cartilage resulting in later degenerative changes. In a cases series by Blackley et al. [2], only one of 59 patients developed osteoarthritis following treatment. In two further studies by Wada et al. [17] and von Steyern et al. [21], each reported one case of osteoarthritis from their series of 15 and 9 patients, respectively. Both patients who developed osteoarthritis were reported to have had an intra-articular fracture either pre- or postoperatively. Suzuki et al. [22] suggested that a lesser amount of residual subchondral bone was associated with a higher incidence of osteoarthritis, reporting 10 cases out of 30 that went on to develop later osteoarthritis. Similar to Wada et al. [17] and von Steyern et al. [21], they reported that the development of osteoarthritis was usually associated with an intra-articular fracture. Presence of an intra-articular fracture was also associated with a higher recurrence rate and need for subsequent reoperation. None of these studies commented on the treatment of the osteoarthritis after it had developed.

There have been few other reports of the treatment of knee osteoarthritis following treatment of GCT. In 2002, Wakitani et al. [23] reported on the use of an Ilizarov procedure for an arthritic knee. Lyall et al. [24] reported on a case of postoperative osteoarthritis who was also treated with a TKR. Both cases reported a good outcome for the respective duration of follow-up. With similar demographics and evolution times, the primary difference between Lyall et al.'s case and our case was that their case utilized allograft bone for the initial treatment of the GCT. They also utilized a tumor prosthesis for the reconstruction indicating that the existing allograft was not strong enough to support primary TKR component fixation. In our patient, the original cementation provided excellent support and allowed us to use a primary TKR implant without stems or augments.

We are not aware of any previous reports of late TKR for the treatment of osteoarthritis when the initial treatment of the GCT lesion was with cementation. Although there is an ongoing debate on the preferred material for filling the void after GCT curettage, possible further reconstructive procedures on the adjacent joint should be taken into consideration when deciding the best treatment option. Given the ease of TKR reconstruction in this patient, we feel that the use of cement to fill the defect after curettage of a GCT lesion is the material of choice. It facilitates the subsequent reconstructive procedure if it becomes necessary in the future and also allows for easier detection of tumor recurrence compared to allograft bone. There is also the theoretical advantage of further tumor ablation by the heat generated at the time of cement polymerization. In this case, the prior cementation allowed us to perform the reconstructive procedure using standard primary implants without the need for stems or augments. Use of navigation could have potentially made the case even easier by avoiding the need for an intramedullary femoral rod. Theoretical contamination of the femoral canal by residual tumor would have been prevented by using navigation though this occurrence would be very unlikely given the negative radiographs and the long duration since initial treatment.

In conclusion, this case illustrates that TKR for the treatment of osteoarthritis following GCT treatment may be facilitated when the primary treatment method is curettage and cementation.

## Figures and Tables

**Figure 5:**